# Epidemic Alphaviruses: Ecology, Emergence and Outbreaks

**DOI:** 10.3390/microorganisms8081167

**Published:** 2020-08-01

**Authors:** Sasha R. Azar, Rafael K. Campos, Nicholas A. Bergren, Vidyleison N. Camargos, Shannan L. Rossi

**Affiliations:** 1 Department of Pathology, The University of Texas Medical Branch, Galveston, TX 77555-0609, USA; srazar@utmb.edu; 2 Department of Microbiology and Immunology, The University of Texas Medical Branch, Galveston, TX 77555-0609, USA; rkkroonc@utmb.edu; 3 Sanofi Pasteur, Swiftwater, PA 18370, USA; nicholas.bergren@sanofi.com; 4 Host-Microorganism Interaction Lab, Department of Microbiology, Institute of Biological Sciences, Universidade Federal de Minas Gerais, Belo Horizonte 31270-901, Brazil; vncamargos@gmail.com; 5 Institute for Human Infection and Immunity, University of Texas Medical Branch, Galveston, TX 77555-0610, USA

**Keywords:** *Alphavirus*, togaviridae, epizootic, enzootic, mosquitoes, outbreaks

## Abstract

Over the past century, the emergence/reemergence of arthropod-borne zoonotic agents has been a growing public health concern. In particular, agents from the genus *Alphavirus* pose a significant risk to both animal and human health. Human alphaviral disease presents with either arthritogenic or encephalitic manifestations and is associated with significant morbidity and/or mortality. Unfortunately, there are presently no vaccines or antiviral measures approved for human use. The present review examines the ecology, epidemiology, disease, past outbreaks, and potential to cause contemporary outbreaks for several alphavirus pathogens.

## 1. Introduction

### 1.1. Overview of Genus Alphavirus

The genus *Alphavirus* is comprised of over thirty viral species that can be divided into eight complexes based on antigenic characteristics [1,2,3]. With the exception of two species, southern elephant seal virus (SESV) and salmon pancreatic disease virus (SPDV), all members of the *Alphavirus* genus are maintained in transmission cycles between specific hematophagous mosquito vectors and susceptible vertebrate reservoirs [1]. In addition to the eight antigenic complexes, the alphaviruses can be divided into two larger groups based loosely on geographic location. The Old World alphaviruses (Sindbis (SINV), chikungunya (CHIKV), o′nyong-nyong (ONNV) and Ross River viruses (RRV)) are found predominantly or originated in Africa, Europe, and Asia and are often characterized as causing febrile illnesses, with rash and debilitating arthralgia as common symptoms. Conversely, the New World alphaviruses (Venezuelan, Eastern and Western equine encephalitis (V/E/W/EEV) and related viruses, Una (UNAV) and Mayaro viruses (MAYV)) are mainly found in the Americas and are largely characterized as causing encephalitic disease. Interestingly, UNAV infections are associated with arthralgia as opposed to encephalitic disease much like other Old World alphaviruses [1,2,3]. Only the alphaviruses responsible for significant outbreaks will be summarized in the present review.

### 1.2. Alphavirus Genome Organization and Replication

Alphaviruses have a single-stranded positive-sense RNA genome approximately 12 kilobases long. The genome possesses a 5′ cap structure and a poly(A) tail. The genome has two major regions: a non-structural domain encoding the non-structural proteins (nsP) on the 5′ terminal portion and a structural domain encoding structural proteins on the 3′ terminal region [3]. The junction between non-structural and structural domains contain a 26S subgenomic promoter for transcription of the subgenomic RNA and the start site for its translation. The non-structural portion is translated as a polyprotein, which is cleaved to produce nsP1, nsP2, nsP3, nsP4 and their other cleavage intermediates. Most alphaviruses have a ‘leaky′ stop codon after nsP3, whereas no stop codon is present in others. The structural domain is translated as a polyprotein from a subgenomic RNA and cleaves into the individual capsid, envelope 3 (E3), envelope 2 (E2), 6K peptide, and envelope 1 (E1) proteins [3].

The replication cycle of alphaviruses is initiated by virion attachment to cells, followed by clathrin-mediated endocytosis leading to internalization of the viral particle. Particle uncoating exposes the viral genome to the cytoplasm [4], which serves as a template for RNA transcription and translation of the non-structural proteins. Virus particle formation then takes place when structural proteins encapsulate the nascent genomic RNA and finally egress by budding from the cells [4].

### 1.3. Alphavirus Structure and Protein Functions

*Alphavirus* virions are enveloped spherical particles with a diameter of approximately 70 nm/700 Å. The particle is comprised of a nucleocapsid core surrounding RNA genome, which encompassed by a host-cell derived lipid bilayer from which envelope glycoprotein spikes are embedded [1,2,3]. The nucleocapsid is comprised of 240 copies of the capsid protein, while the viral envelope proper is comprised of 80 trimeric glycoprotein spikes, with each spike consisting of three E1-E2 heterodimers [2,3]. Both E1 and E2 have discrete functions in the virus life cycle, with E2 serving as the receptor binding domain, while E1 is associated with host membrane fusion. The E3 protein is a cleavage product from the proteolytic processing of the pre-E2 (pE2) after pE2 association with E1 to form glycoprotein spikes, allowing for spike maturation [1,2,3]. The 6K protein similarly acts as a leader peptide for E1, and is reported to enhance viral egress from infected cells via the formation of membrane pores or channels [1,2].

The non-structural proteins, unlike the viral genome and the structural components of the virion, are not incorporated into progeny virions [2]. These proteins are involved in the replication of the viral genome and assembly of progeny virions. Of these, nsP1 has both methyltransferase and guanylyltransferase activities, which are required for the capping of genomic and subgenomic RNA transcripts [1,3]. nsP2 is the largest of the alphaviruses nsPs and has among the most functions; the N-terminal end has nucleoside triphosphatase activity and also serves as the viral helicase, while the C-terminal end serves as the viral protease responsible for processing the nsP polyprotein [1,3]. The exact functions of nsP3 are presently unclear, although it is known that it is a phosphoprotein and contains Src homology 3 (SH3) binding domains in the C-terminal region [1]. Finally, the nsP4 protein is the RNA-dependent RNA polymerase responsible for the replication of the viral genome [1,3].

## 2. Specific Alphaviruses

### 2.1. Venezuelan Equine Encephalitis Virus (VEEV)

#### 2.1.1. Disease

Venezuelan equine encephalitis virus is the etiologic agent for both human and equine Venezuelan equine encephalitis (VEE). VEEV was first recognized as an equine disease during the 1930s [5], and later identified as a human pathogen in the 1940s [6,7]. In humans, VEEV has an incubation period of 1–5 days, which is nearly always followed by rapid onset of symptoms including fever, headache, myalgia, ocular pain, nausea, back pains, vomiting and diarrhea [8,9,10]. This initial clinical picture is similar to that of many viral infections, but in 4–14% of the cases, it can progress to a more serious encephalitic disease characterized by photophobia, confusion, seizures, convulsions, stupor, behavioral changes, alterations of consciousness, unilateral paralysis and coma [11,12,13]. Laboratory exams may show leukopenia, lymphopenia and elevated transaminases [14], renal failure [13], and cerebrospinal fluid may show elevated proteins and increase in lymphocyte counts [14]. The case fatality rate in adults is lower than 1% and varies based upon the viral subtype, but serious neurological disease can occur in up to ~15% of infected people [11,13,15]. Interestingly, young children have a higher disease burden and fatality rate [16,17]. Pregnant women with VEE are at risk of abortions, preterm deliveries, congenital disabilities and stillbirths [18]. Infection with VEEV can also cause sequelae in survivors, and 25% of the hospitalized patients were reported to have long-lasting neurological problems, including headaches, severe fatigue and depression [19,20]. Humans are susceptible to infections with both enzootic (constantly circulating in nature) and epizootic (epidemic or outbreak) strains [9]. The majority of VEEV infections occur following the bite of an infected mosquito. However, the virus is highly infectious by the aerosol route and manipulations with samples containing the virus may result in virus aerosolization. In laboratory settings, most accidential VEEV infections occur from inhaling aerosolized particles [17].

VEEV equine infection results in a range of clinical manifestations from inapparent infection and seroconversion to systemic disease with lethargy, fever, tachycardia, depression and anorexia to signs of clinical encephalitic; oftentimes detectable VEE in horses is fatal [11,13,15,21]. Animals infected with enzootic strains usually do not develop viremia or disease [21,22]. This difference appears to be largely mediated by the specific sequence of the envelope protein of epizootic strains. In experimental infections with viruses containing the envelope proteins of an epizootic virus, most horses exhibit fever within 36 h of virus infection, followed by lethargy and anorexia. Approximately 75% of the horses develop clinical signs of encephalitis starting at days 6 to 8 post infection [21]. Outbreaks caused by VEEV have widely varying case-fatality rates for equines, with the strains usually not being lethal and epizootic strains killing 20–80% of infected animals [17].

Although there are no vaccines approved for use in the general human population, a live-attenuated vaccine created by serial passage of the TrD strain, TC-83, has been used to immunize researches working with the virus and members of the armed forces. This Investigational New Drug (IND) vaccination provides immunity to approximately 80% of the vaccinated individuals [23]. However, TC-83 vaccination is also known to result in various side effects, which are usually a more mild version of the signs and symptoms caused by VEEV infection but can still be severe, including fever, headache, malaise and photophobia [24]. Presently, there are no drugs or antivirals to treat VEE in humans.

#### 2.1.2. Ecology and Transmission Cycles of VEEV

The VEE antigenic complex of viruses includes six antigenic subtypes (I to VI) each including one or more species (I. Mosso das Pedras virus and VEEV; II. Everglades virus; III. Mucambo and Tonates viruses; IV. Pixuna virus; V. Cabassou virus; VI. Rio Negro virus). Little is known about the ecology and cycle of most species in the VEE complex, with the exception of VEEV, for which there is more information available. Most species are present in South America, with the exception of Everglades virus, which is only found in Florida in the United States [22]. Tonate virus, Everglades virus and VEEV have been reported in North America, while VEEV is the only species described in Central America as well [22]. While VEEV has been shown to cause disease in horses, the host range for other species of the VEE complex is largely unknown [22]. Everglades virus is known to infect and circulate in rodents and mosquitoes, and serological evidence suggests it can also infect dogs and humans [25]. Mucambo virus is also known to cause disease in humans, but to date has not been reported to cause large outbreaks.

Subtype I is implicated in numerous equine and human epidemics. VEE antigenic subtype I is divided into epizootic subtypes IAB and IC, and well as enzootic subtypes ID and IE. It has been found in multiple locations across the Americas; the most common epidemic viruses found belong to the subtype IAB, and have been isolated in South, Central and North America; IC has been identified in South America; ID is found in South and Central America; and IE is located in Central and North America [22]. VEEV preferentially utilizes mosquitoes in the subgenus *Culex* (*Cx.*) (*Melanoconion*) [22,26] as vectors. It has been postulated nevertheless that the most widespread outbreaks may involve adaptation of the virus to mosquitoes of the species *Aedes (Ae.) Ochlerotatus taeniorhynchus* [15,27]. Other probable vectors for VEEV in which the virus has been recovered from mosquitoes or shown to be inflectable in the lab include the genera *Haemagogus* [28], *Deinocerites* [28], *Wyeomyia* [28], *Mansonia*, *Psorophora* [29,30,31,32], and *Anopheles* [33,34].

VEEV is transmitted in enzootic and epizootic cycles. Large human outbreaks are usually associated with an extensive number of equine infected by an epizootic strain of VEEV [22], which become viremic allowing *Ae. Ochlerotatus taeniorhynchus* mosquitoes to become infected and act as vectors from horses to humans. The VEEV enzootic subtypes are ID and IE, whereas IAB and IC are epizootic [22]. This appears to be largely determined by the equine virulence of the virus, which influences their ability to cause high level viremia, as epizootic strains are more virulent and able to cause higher levels of viremia than enzootic strains [22]. The VEEV cycle in nature is maintained by enzootic cycles in rodents [35,36,37], mosquitoes, and possibly other wild animals such as bats [38,39,40,41]. Forest-dwelling mosquitoes, mainly of the *Culex* genus [30], are vectors and infect wild animals to maintain the enzootic cycle. Natural reservoirs for VEEV are proposed to be rodents of the subfamily *Sigmodontinae* [35,42]. Several other potential hosts have also been described by experimental infection in a lab, report of natural infection or serology of wild animals, including other rodents [37,38], bats [38,39,40,41], wild swine [43], deer [43], rabbits [44], and opossums [42]. These hosts also have potential to act as reservoirs and more field studies in areas with VEEV are necessary to establish the contribution of each host to its enzootic cycle.

#### 2.1.3. Outbreaks

VEEV outbreaks have occurred episodically from enzootic progenitors over the last century. As a result, VEEV strains of the IAB and IC disappear from the human and equid populations after the epidemic subsides. Epizootics are initiated when IAB or IC VEEV infect horses, producing high titer viremia that efficiently infect feeding mosquitoes [17,45]. Unfortunately, outbreaks in equine populations have resulted in death up to case-fatality ratios as high as 80% and are oftentimes the first indicator of circulating epizootic VEEV [17]. The outcome of equine infection is at least partially controlled by mutations in the viral envelope glycoproteins [21,46,47], although other viral mutations and host factors are also likely to play a role.

VEEV was first isolated from the brain of an infected equine from Venezuela in 1938 [5]. It is possible that VEEV outbreaks may have started as early as the 1920s before they were widely recognized [22,48]. The 1943 outbreak in Trinidad resulted in recognizing VEEV as a human pathogen [6]. The prototype IAB VEEV strain, Trinidad donkey (TrD), was isolated from the brain of a donkey at that time [6]. VEEV outbreaks then occurred episodically, with periods of approximately 10 years in between major outbreaks [6,13,22,49,50,51,52,53,54,55,56]. The 1960s had several large and devastating outbreaks across South America. A large outbreak occurred in 1962 that started in Columbia and spread to Venezuela, in which there were over 25,000 cases of human infection with 176 fatalities [16]. Cases continued in Colombia between 1967 and 1968. Closely thereafter in 1969, Ecuador would report approximately 20,000 equine deaths and 31,000 human cases of disease resulting in 310 deaths [16]. The virus spread northward during that year to El Salvador, Guatemala and Mexico [57]. The disease burden in these countries exceeded 50,000 human cases and 50,000 fatal equine infections [57]. VEEV infections continued to spread northward and by 1970–1971, the outbreak crossed into the United States. Texas would report more than 1500 horse deaths, 110 human cases and hundreds of VEEV isolations from field-caught mosquitoes [57,58]. A massive vaccination campaign to halt further spread and devastation was employed using TC-83 to great effect [59,60]. By 1972, the outbreak had subsided.

Less than a decade later, a large outbreak occurred in 1979 in Mexico and Texas [42], during which tens of thousands of people were infected [42]. No reports of VEEV epizootics were reported in the 1980s. In 1995, however, the largest recognized human outbreak was observed in Venezuela and Colombia in 1995 [50,61] in which approximately 100,000 people and 50,000 equids were infected. More recent outbreaks occurred in 2005 and 2006 in Peru [52]. Over 20% of the people of Iquitos, Peru, had neutralizing antibodies against VEEV, suggesting widespread infection in the city [52]. During these two years, there were approximately 100 confirmed infections and two deaths were reported [52]. Because the VEEV clinical picture is indistinguishable from that of other common arboviruses, cases are likely to be frequently misdiagnosed as being other more common arboviruses, since the molecular or serological diagnosis is rarely done [16].

#### 2.1.4. Potential for Future Outbreaks

Based on the episodic timing of VEEV outbreaks in the past, outbreaks of VEEV will almost certainly happen. To predict and understand the characteristic of these outbreaks, it is paramount that additional studies are done to understand the interaction of VEEV with different hosts, vectors and reservoirs, as well as virus evolution and phylogenetics. Several mechanisms were proposed for the emergence of VEEV epidemics. The most prominent hypothesis is that periodic evolution of IAB and IC VEEV strains facilitate for these epizootics. This is supported by phylogenetic studies [62,63] and laboratory experiments demonstrating that point mutations are able to cause changes in virulence and determine whether the virus can establish viremia in equines [21,46,47]. High level viremia in equines (10^6^–10^8^ plaque forming units (PFU)/mL) is an important factor for epidemic potential, as it results in many new mosquito infections in the region [45]. Vector competency is another aspect that may profoundly influence the chances of new outbreaks of VEEV. Although *Culex* (*Melanoconion*) is the main vector for VEEV during the enzootic cycle and *Ae. Ochlerotatus taeniorhynchus* is the main vector during the epizootic cycle there is evidence that many other mosquito species could be competent vectors and may yet become important natural vectors once new adaptive mutations arise or mosquito distribution expands [27].

To predict and prepare for future outbreaks, it is important to determine mutations affecting vector competence as well as viremia in the hosts. Mutations in the envelope glycoproteins, for example, have been linked to changes in both of these parameters, and could be used to monitor viral population and determine whether epizootics are likely to occur. Monitoring virus in mosquitoes, as well as viremia in equines and wild animals, could also be an effective method to predict the imminence of an outbreak. Development of new therapies and safer and more effective vaccines are also key strategies to prepare for future outbreaks and protect against a potential deliberate release of the virus.

### 2.2. Eastern Equine Encephalitis Virus/Madariaga Virus (EEEV/MADV)

#### 2.2.1. Disease

Eastern equine encephalitis virus is the causative agent of Eastern equine encephalitis (EEE). The EEEV antigenic complex is comprised of four phylogenetically distinct lineages, one of which is found circulating in North America and the Caribbean, with the remaining three circulating in South and Central America [64,65]. Until 2013, the entire EEEV antigenic complex was referred to as EEEV, with North American lineage strains referred to as NA EEEV (Lineage I) and strains from any of the three South American lineages referred to SA EEEV. In 2013, the International Committee on Taxonomy of Viruses (ICTV) approved a proposal to reassign the three South American lineages (Lineages II, III, and IV) of EEEV to the newly created *Alphavirus* species Madariaga virus (MADV) based on differences in genetics/evolution, ecology, epidemiology, and virulence [65,66]. These distinctions will therefore be highlighted as appropriate over the course of this section of the review.

Human infection by North American strains of EEEV is thought to be frequently asymptomatic, with some reports estimating that up to 96% percent of patients demonstrate no symptoms of infection [17,64,67,68,69,70]. In patients who will demonstrate symptoms, a 4–10-day prodromal phase follows the bite of an infected mosquito, characterized primarily by nonspecific symptoms, including but not limited to fever, chills, malaise, and myalgia. This nondescript febrile illness can either resolve, or progress to encephalitic disease characterized by fever, headaches, nausea, vomiting, diarrhea, altered mental status, seizures, and coma [17,68,71,72]. Despite its low apparent:inapparent ratio of 1:23 [64,70,72], symptomatic infection is associated with high mortality rates, with estimates of between 30–70% depending on the outbreak [17,64,67,68,71,72,73], and up to 50% of survivors demonstrating serious neurological sequelae such as intellectual impairments, psychiatric manifestations, seizure disorders and nervous palsies [68,73,74,75,76,77,78]. Anatomically, EEEV infection is particularly associated with lesions in the basal ganglia and thalamus based on MRI and/or CT imaging, although brain stem lesions have also been described [79]. There is some evidence to suggest that symptomatic infection by EEEV is more fatal in adults than children, although surviving adults demonstrate milder sequelae [64,72,73,80]. In stark contrast, MADV (reported in the literature as SA EEEV until 2015) has historically been considered an equine pathogen [64,72,81]. Indeed, until an outbreak taking place in Panama in 2010, MADV has been associated with only two cases of fatal human disease, one taking place in Brazil in 1956, and the other in Trinidad in 1972 [82,83,84,85]. Seven confirmed human patients were characterized over the course of the 2010 Panama outbreak, with only one reported fatality, which was not confirmed to be MADV). All seven patients progressed to neurologic disease, with six of the seven demonstrating seizures that progressed to status epilepticus (86% vs. 25% observed in North American outbreak) [79,85]. Imaging analyses also demonstrated differences from EEEV, with temporal lobe lesions observed as opposed to the characteristic basal ganglia and thalamic lesions previously reported [79,85]. Serosurveys conducted in endemic regions of Peru and Panama have demonstrated that 2 to 5% of the human population in these areas have had prior exposure to MADV, suggesting a high rate of asymptomatic or subclinical infections [39,84,85].

Equine infection by either EEEV or MADV are oftentimes serious; EEEV has a case-fatality of 80–90% in equids [64,67,86] and MADV has been demonstrated to have between 70–90% case-fatality, depending on the individual outbreak [85,87]. Clinical disease in equids generally follows an incubation period of 1–3 weeks following the bite of an infected mosquito [67], and initially manifests as a short-lived, high fever (up to 106 °F). Subsequent signs and symptoms can include manifestations such as ataxia, inappetence, blindness, and depression [64,67,86,87,88,89,90]. Terminal disease is often characterized by convulsions, loss of consciousness, loss of autonomic functions and death [64,67].

#### 2.2.2. Ecology and Transmission Cycles of EEEV and MADV

EEEV naturally circulates in an enzootic transmission cycle between its principle vector *Culiseta (Cs.) melanura* and birds (largely passerines) located in freshwater hardwood swamps [64,65,72]. With respect to North America, this results in regional variations in transmission based on climate and season. In the temperate climates of the Northeastern United States, transmission is seasonal, with detection of viremic birds taking place in the spring (May and June), preceding the detection the virus in mosquitoes, which typically takes place in the mid-summer months. Enzootic circulation of EEEV will persist until the onset of sustained cold weather in November or December [64,91]. However, in the Southeastern regions of the United States, such as Florida, enzootic transmission of EEEV is maintained year-round [64,92,93]. Likewise, MADV in South and Central America is transmitted continuously throughout the year [64,91]. It is important to note that the enzootic cycle of MADV is largely restricted to areas deep within rainforests, which makes full characterization of the enzootic cycle logistically challenging. It is understood, however, that unlike North American EEEV, the primary vector for MADV are *Cx.* (*Melanoconion*) mosquitoes [64,91].

Both enzootic and epizootic cycles for EEEV in North America are well-characterized. The definitive mosquito vector for EEEV is *Cs. melanura*. Some studies have recognized that alternate mosquitoes such as *Cx. restuans* and *Cx. erraticus* can serve as enzootic vectors in some southeastern foci where *Cs. melanura* are not present or present in smaller numbers [72,94]. The enzootic cycle is largely limited to freshwater hardwood swamps or other such wooded wetlands, primarily due to the habitat requirements required for development of *Cs. melanura* larva [64,91,95,96]. *Cs. melanura* are primarily known to be ornithophilic, with multiple field analyses conducted on bloodmeals of engorged females demonstrating a pronounced avian host preference [91,93,97,98]. Specifically, both serological and bloodmeal-based methodologies have incriminated a distinct host preference of *Cs. melanura* for passerines (perching or songbirds) [64,91,97,99,100,101,102]. Experimental infection studies in various passerines [103,104,105], as well as detection of anti-EEEV antibodies passerines during serological surveys [103,106,107] have conclusively incriminated birds of this order as the natural vertebrate reservoir for EEEV. Nonetheless, it is worth noting that experimental studies have demonstrated the capacity for EEEV to infect a myriad of birds, including Galliformes such as turkeys, chickens, and pheasants [108,109,110], Pelecaniformes such as ibises and egrets [103,111], Anseriformes such as ducks [112], and Columbiformes such as pigeons [104]. Given the proclivity of *Cs. melanura* for feeding on passerines [64,91,97,99,100,101,102], coupled with high titers (up to 9 log_10_ PFU/mL) observed in experimentally infected passerines [105], non-passerines likely do not play a significant role in the enzootic ecology of EEEV.

Enzootic transmission of EEEV is sustained by infection of passerine birds, specifically, by infection of hatching year chicks, likely due to both a lack of pre-existing immunity and decreased propensity to utilize anti-mosquito behaviors to avoid bites [64,106]. Viremia in hatching year chicks is also of greater magnitude and duration allowing for efficient infection of *Cs. melanura* [64]. It is worth noting that anecdotal evidence indicates the virus can be transmitted from infected to uninfected gamebirds through behaviors such as pecking, preening and cannibalism, although the role this plays in EEEV ecology, if any, is unclear [64,67]. The transition from enzootic circulation to an epizootic outbreak is generally thought to require the involvement of mosquitoes that are more promiscuous is their host choice, exhibiting both mammophilic and ornithophilic tendencies; these mosquitoes are commonly referred as “bridge vectors”. For EEEV transmission cycles, the types of mosquitoes that serve as bridge vectors can differ on regional species abundance and host availability although *Ae. vexans*, *Ae. solicitans*, *Ae. canadensis*, and *Coquillettidia perturbans* have been incriminated in this role [72,78]. Bridge vectors will become infected by feeding on viremic birds, and subsequently can infect both avian hosts and mammalian dead ends. It is largely through these bridge vectors that humans and equids (which are both dead end hosts for EEEV) are infected [64,72]. It is also worth noting that in some regions, it appears that *Cs. melanura* also acts as the epizootic vector [72,113].

In contrast, the MADV enzootic transmission cycle remains less clear. This is at least in part due to difficult to reach foci deep within tropical rainforests [64,91]. What is known is that enzootic transmission, like EEEV in southern foci in North America, is maintained near continuously year-round [64,91]. The vast proportion of MADV isolations have been from *Cx*. (*Melanoconion*) mosquitoes, leading to regional representatives of this subgenus being incriminated as the primary enzootic vectors of MADV [64,91]. Specific representative examples of enzootic *Cx*. (*Melanoconion*) vectors include *Cx. taeniopus* (Panama and Brazil), *Cx. dunni*, and *Cx. panocossa* (Venezuela), and *Cx. pedroi* (Peru) [64,91]. Experimentally, a field population of *Cx. pedroi* from Peru has been demonstrated to be efficiently infected when exposed to ≈5 log_10_ PFU/mL of Lineage III (a lineage generally localized to the northeastern parts of South America) EEEV/MADV [64,91,114]. In addition to their roles in the enzootic transmission of MADV, these *Cx*. (*Melanoconian*) can potentially also serve as epizootic vectors [64,65,91,115,116,117]. However, it is worth noting that field evidence does indicate that *Cx*. (*Melanoconian*) do not tend to move outside their rainforest niche [118], indicating at least some role for potential bridge vectors. Furthermore, the vertebrate reservoir is unknown. Potential sources include birds, rodents, marsupials and reptiles [64,65,91], all of which are hosts that are fed on by various species of *Cx*. (*Melanoconian*) [119]. Experimental studies have demonstrated that both birds and rodents become viremic and could potentially serve as reservoirs for MADV [120]. Strikingly, during a MADV epizootic in Panama in 2010, a small subset of both human and equine subjects were found to be co-infected with both VEEV and MADV, in part providing some support rodents serving as potential reservoirs for MADV [54,85].

#### 2.2.3. History of Outbreaks

Evidence indicates that EEEV has caused outbreaks in North America in equines as early as 1831 [90,121]. The causative agent was ultimately isolated from the brain of an infected horse in New Jersey during a large scale (>1000 horses), multistate (Maryland, Delaware, Virginia, New Jersey) epizootic outbreak in horses in 1933 [64,121]. Subsequently, additional equine epizootics took place in the states of Virginia and North Carolina in 1934 and 1935, respectively, during which the role of birds in the ecology of EEEV was first proposed [64,121,122], although this would not be confirmed until 1950, when the virus was isolated from the blood of a seemingly healthy purple grackle in Louisiana [64,121,123]. The largest reported epizootic took place in southern Louisiana and eastern Texas in 1947 with an estimated 14,334 infections in horses and mules and over 11,000 deaths [64,124]. Human infection by EEEV was confirmed in 1938, when the virus was isolated from post-mortem brain samples taken from a fatal pediatric case in Boston [64,121,125]. Since then, multiple outbreaks in the eastern United States have occurred, with 112 human cases identified between the years of 1938–1965, 99 cases between 1961–1985, 121 cases between 2003–2016, and 49 cases from 2017 to present [64,68,76,121,126,127]. Human cases vary from year to year, typically with 15 or fewer yearly cases [64,68,76,121]. Since the inception of statewide reporting of arbovirus infections to ArboNET began in 2003, at least a single case of human EEEV infection has been documented per year [68,126,127]. Some years have more reported cases, including Massachusetts in 1938 (34 human cases, 248 equine cases) [64,75,80], New Jersey in 1959 (32 human cases, 66 equine cases) [64,70,73], and across multiple states in 2019 (38 human cases) [69,76,78].

Epizootics of MADV were reported as early as 1908. Although the virus was isolated in horses in Argentina in 1930, it not identified as South American EEEV/MADV until 1953 [64,128]. Until recently, MADV epizootics were primarily only in equines [64,128,129]. Prior to 2010, only three cases of human infection with SA-EEEV/MADV had been reported in the literature [83,85]. Of the 19 hospitalized patients diagnosed with encephalitis in Panama in 2010, seven of these patients were confirmed to be infected with MADV and one patient was co-infected with MADV and VEEV. Community based surveillance identified 99 additional patients with alphavirus infection, of which 13 were found to be infected with MADV and one to be infected with both MADV and VEEV [85]. Interestingly, this epizootic also had 50 equine infections [85]. From 2015–2016, eight MADV infections were reported in a cohort of young school children in Haiti [130,131]. Similarly, in 2016, a single adolescent patient in Venezuela was diagnosed with MADV after demonstrating signs of a nonspecific febrile illness that was hypothesized to be due to recent travel to a locale that reported an equine epizootic of MADV [132]. Despite this increased attention, population level serosurveys have repeatedly demonstrated low percentages of neutralizing antibodies against MADV in various human populations [84,128,129,133].

#### 2.2.4. Potential for Future Outbreaks

EEEV and MADV maintain annual patterns of seasonal or sustained transmission. With respect to EEEV, a small number of veterinary and human cases have been reported yearly since tracking began in 2003 [127]. However, the multistate (Massachusetts, Michigan, Connecticut, New Jersey, Rhode Island, North Carolina, and Tennessee) epizootic outbreak in 2019 was among the largest epizootic EEEV outbreaks with human cases [68,78]. The reason for this sudden uptick is still unknown and highlights the unpredictability of epizootics. The frequency and scale of these outbreaks is likely to be directly affected by weather, as a climatological analysis in Massachusetts has demonstrated that heavy rainfall in the preceding year has been associated with EEEV outbreaks/epizootics [64,134].

Given growing urbanization driving increased human contact with previously undisturbed biomes, global trade/travel driving the spread of invasive mosquitoes and viruses outside their historical sites, and climate change, it is likely that both EEEV and MADV will continue to present threats to human and equid health in the coming years [135,136,137,138]. This is particularly concerning due to the potential introduction of invasive mosquitoes that can serve as efficient bridge vectors for either virus. Vaccines to prevent disease in humans are currently unavailable. Equine vaccination is effective at preventing EEE, although evidence indicates there are windows of susceptibility following vaccination [64,121]. Arbovirus surveillance is a critical tool in the abatement of EEEV and MADV outbreaks.

### 2.3. Western Equine Encephalitis Virus (WEEV)

#### 2.3.1. Disease

Western equine encephalitis virus is the etiologic agent of Western equine encephalitis (WEE). WEEV infection can result in a broad spectrum of disease outcomes ranging from subclinical, febrile symptoms, to encephalitis/encephalomyelitis [139]. The ratio of inapparent infection to disease symptoms is dependent on age and is skewed strikingly toward infants: 1:1 in infants less than one-year-old, 58:1 in children between one and four-years-old, and 1150:1 in people greater than 14-years-old [140]. Signs and symptoms manifest suddenly and include fever, chills, headache, nausea, and vomiting [139]. After initial onset of illness, neurological signs and symptoms, including lethargy, drowsiness, neck stiffness, photophobia, vertigo, and mental status changes can manifest within a few days [141]. Infants are particularly prone to irritability, convulsions, upper motor neuron deficits, and tremors, though these signs can appear in older patients as well [142]. Case-fatality ranges between 3 and 15% [139,143]. Neurological sequelae is often seen in patients recovering from neurological complications associated with the disease and is also skewed toward younger individuals [144,145].

Disease in equids is generally more severe than humans with case fatality rates ranging from 10 to 50% [139]. Following a one- to three-week incubation period, febrile signs will manifest early in the disease progression including fever, anorexia, restlessness, irritability, decreased movement, and ataxia [139]. Subsequent neurological involvement can manifest as head pressing, stupor, head drooping, blindness, flaccid lips, involuntary movements, inability to stand, partially closed eyelids, convulsions, and paralysis. Terminally the animal can exhibit involuntary eye movement, paddling, and coma [146]. A formalin-inactivated vaccine against WEEV is available for purchase and is routinely used to immunize horses against infection. Unfortunately the vaccine has low immunogenicity and requires annual boosters, though adequate coverage for affected areas can be achieved [147].

#### 2.3.2. Ecology and Transmission Cycles of WEEV

WEEV typically follows an annual pattern of emergence during the spring, summer maintenance, decline during the fall, and quiescence in the winter months [139]. The primary summer transmission cycle of WEEV in North America is well understood with *Cx. tarsalis* acting as the primary vector and passeriform birds acting as amplification [140,148,149,150]. Horses and humans are considered dead-end hosts.

Nestling house finches, sparrows and other nestling passerines amplify the virus during the spring [140,151,152,153]. The means by which the virus enters this amplification cycle remains unclear; studies in Texas and Colorado indicate that nestling passerines become infected before *Cx. tarsalis,* thus implicating some unknown sylvatic vector [152,153], though, initial amplification in nestling passerines as a means of emergence is not found in all regions [140]. Furthermore, in the northern latitudes WEEV has been found actively circulating in snowshoe hares and ground squirrels before *Cx. tarsalis* terminates diapause indicating a sylvatic transmission cycle precedes the springtime amplification, possibly vectored by mosquito belonging to the *Aedes* genus or *Cs. inornata* species [154,155,156,157,158]. Adult passerines play a greater role in transmission during the summer. Additionally, domestic Galliformes (chickens) and Columbiformes (doves) may contribute to virus maintenance and amplification, though their ability to amplify the virus is dependent on their age, which makes them poor hosts [140,153]. Human and equine incidental infections are identified during the summer after transmission exceeds some undefined minimum threshold. Other ornithophilic mosquitoes may also participate in transmission, though their significance to virus maintenance is limited due to them being poor vectors [139]. As the year persists, the feeding pattern of *Cx. tarsalis* becomes increasingly catholic. As the season progresses, virus transmission can spill over into an *Aedes* spp.-mammal cycle. WEEV infections begin to subside in the colder fall months with concurrent lower detection rates in *Cx. tarsalis* as they begin to die off for the year [139,158,159,160]. Usually few infections occur after October [140,143]. The mechanism by which WEEV overwinters remains cryptic. Significant work has been undertaken to determine potential overwintering mechanisms; however, no firm conclusions could be made [149,161,162,163,164,165,166].

There is some evidence that ponies and burrows develop sufficiently high viremias to infect mosquitoes [167,168,169], though the extent to which equids participate in the transmission of WEEV, if at all, remains unknown. Even though low proportions of mosquitoes would become infected from viremic horses, the large number of horses in North America in the early 20th century, for agricultural use, could potentially compensate for the low proportions.

Over the last century, WEEV enzootic activity and WEE cases have significantly declined. WEEV has not been detected among its natural enzootic host and vector species since a house sparrow tested seropositive in Harris County, Texas, in 2015 [170] and a mosquito pool confirmed PCR-positive in Clark County, Nevada in 2013 [171]. Furthermore, a recent study tested a large number of *Cx. tarsalis* mosquito pools from Larimer County, Colorado, and found no evidence of WEEV [172]. The specific reason WEEV has apparently ceased its enzootic transmission and disease is something of a mystery, though reduction in incidence does correlate with a drastic decline in the US horse population and increased vaccination coverage of horses [139].

#### 2.3.3. History of Outbreaks

WEEV has caused numerous documented epizootic events during the 20th century. The epizootic that prompted the discovery of the agent occurred in 1930 where approximately 6000 horses were infected with a 50% case fatality rate [173]. Subsequently severe outbreaks appeared to progress toward the east from California during the years of 1931 through 1934, reaching the grasslands of Canada in 1935 [139]. Further, several severe outbreaks occurred during the mid-1930s and throughout the 1940s [174]. More equids than humans exhibit WEEV disease (due to the increased amount of exposure to *Cx. tarsalis* and equids exhibiting more severe symptoms than humans) during epidemics though the specific intensity of each epidemic can vary widely. For example, during 1937 and 1938 more than 300,000 horses were infected with WEEV [175]. An especially severe epidemic that occurred in 1941 resulted in 3336 human deaths in the United States (mostly in the Dakotas, Minnesota, and Nebraska) and Canada (Manitoba and Saskatchewan) [175,176].

After the 1930s and 1940s, the number of human and equine cases began to decline. One well-studied epidemic that occurred in 1952 in the Central Valley of California resulted in 375 cases of WEEV-induced human encephalitis, nine of which resulted in death. Other epidemics occurred in 1958, 1965, and 1975 [177,178]. Some of the most severe epizootic events occurred in Midwestern North America and southern Manitoba, for example, in 1941. Additionally, several epidemics occurred in Midwestern North American and southern Manitoba during the late 20th century with the 1975 epidemic causing 53 human and 420 equine cases [179]. WEE continued to occur sporadically throughout the 1980s and 1990s with the last reported human case in North America occurring in 1998. One fatal case of WEE arose in Uruguay during April of 2009 in an otherwise healthy 14-year-old boy [180]. The infecting virus appeared to show a close phylogenetic relationship with the avirulent WEEV/Imperial181 isolate, though more intensive phylogenetic techniques might be needed to more closely define this similarity.

#### 2.3.4. Potential for Future Outbreaks

Given the reduction in WEE disease and WEEV enzootic circulation, understanding whether it is able to emerge is inexorably linked to a proper understanding of its “submergence”. A particularly useful means of understanding the submergence of WEEV is to observe the evolution of the virus over time. [181]. Isolates of WEEV appear to reduce in virulence overtime in murine models [182,183]. However, studies where reductions in virulence or enzootic fitness are explained temporally by choosing representative isolates from each decade within the 20th century does not factor in the evolutionary pattern of WEEV over the 20th century [182,184,185].

Phylogenetically, WEEV clusters into two main groups: Group A and Group B [186]. Group A viruses became extinct with the emergence of the Group B lineage around 1946 [181]. Furthermore, Group B viruses subsequently underwent a pattern of emergence and purification resulting in the subgroups B1, B2, and B3. This pattern persisted to the point where after 1993, Group B3 was the only circulating linage attendant with a putative reduction in population size [181]. Virulence for Group A viruses may be partially explained by a glutamine residue on position 214 of the E2 glycoprotein, when this residue is replaced with an arginine, the residue present on Group B viruses, a reduction in murine neurovirulence is observed [187]. Group A viruses typically have multiple laboratory amplification cycles (passages) in suckling mice or some other murine passage model due to their age and the standard virological practices at the time of their isolation. A direct result of numerous passage history of Group A viruses is the inability to determine whether the E2-Q214R mutation arose in nature or passage in murine models. Furthermore, the E2-Q214R mutation is not the sole determinant of virulence as several Group B1 isolates are highly virulent with their residue at E2–214 being an arginine [181]. Group A viruses are also less fit in *Cx. tarsalis* than Group B viruses [187].

Given the evolutionary pattern of WEEV and passage history of many of the isolates, conducting experiments with representative isolates from each decade has not provided clear reasons for WEEV′s submergence [182,184,185]. However, upon phylogenetic analysis six non-synonymous, synapomorphic mutations, which define the Group B1, B2, and B3 clades, were defined and indicated as potential for positive selection [181]. Reverse genetic studies were then conducted in which the past residues were cloned into a contemporary Group B3 isolate, Imperial181 [188]. These mutations were assessed for their effect on virulence in the hamster model and enzootic fitness in house sparrows (HOSP) and *Cx. tarsalis*. The mutations had no effect on virulence and the past mutations resulted in reductions in fitness in HOSPs and *Cx. tarsalis*, that is wild-type Imperial181 was more fit in HOSPs and *Cx. tarsalis* [188].

The reverse genetic and phylogenetic data paints an odd picture where WEEV circulation has reduced in nature, with the viral population having undergone reductions and become less diverse, and yet evolved to become more fit in enzootic hosts and vectors. The specific factors that precipitated this evolutionary trajectory are ultimately unknown. Certainly, this putative downward evolutionary trajectory was precipitated by specific ecological events, though they remain cryptic. Hypotheses include horses participating to some extent in the transmission and their reduction over the 20th century removed an important amplifying host; improved vector control methods for *Cx. tarsalis*, though transmission of West Nile virus is facilitated through this vector currently, or some other relevant changes in the enzootic hosts or vectors.

The specific reason for WEEV′s submergence may never be completely understood. Though it does provide valuable insight regarding the significance viral ecology has on the health of the viral population. Because the specific ecological factors that precipitated WEEV′s submergence are not understood, it is unclear whether the virus is capable of reemerging. However, this scenario remains unlikely due to the reductions in virulence seen in the Group B viruses and near non-existent detection of the virus in nature, though it cannot be completely ruled out.

### 2.4. Chikungunya Virus (CHIKV)

#### 2.4.1. Disease

Chikungunya virus is the etiologic agent of chikungunya fever (CHIKF), a rapid-onset arboviral disease characterized primarily by debilitating arthralgia. The percentage of those infected who are symptomatic is higher than for other arboviruses [189], allowing the identification of cases to be relatively easier than other infections with more asymptomatic cases. Following an incubation period of 3–6 days, CHIKF occurs rapidly with greater than 90% of patients experiencing fever, myalgia and arthralgias, particularly in the extremities such as feet and hands [190]. Rash can also be seen in about 40–50% of patients. High titer viremia, which can approach 10^9^ PFU/mL, is roughly concurrent with symptom onset and lasts from 5–7 days [190,191]. The antibody response also begins in the first week of acute infection and neutralizing IgG is believed to provide life-long immunity to second infection. Typically, CHIKF is self-limiting and acute disease resolves within two weeks.

Not all populations are affected similarly with the elderly and immunocompromised being more susceptible to severe disease. Fortunately, the case fatality rate for CHIKF is extremely low, especially among young adults without co-morbidities; the Pan American Health Organization (PAHO) reported only 101 deaths in over 123,000 confirmed cases in 2017 [192]. This rate is similar to the approximately 0.1% rate reported in other outbreaks and areas [193]. For the older population, or those with underlying health concerns, the case fatality rate rises significantly and mortality correlates with disease severity [193,194]. Likely in part due to the large number of worldwide CHIKV infections, rare complications and unusual disease outcomes during CHIKF have been observed. For example, neurological disease may not be as rare as initially thought; encephalitis, myelitis, Guillain-Barré syndrome and ocular involvement including uveitis and retinitis have been reported [195,196,197]. Mother-to-fetus perinatal transmission was also observed [198].

The most concerning part of CHIKF, perhaps, is the duration of sequelae, which can last from several months to several years. Persistent arthralgia was most often reported in the peripheral joints such as hands, ankles and knees (summarized in [199]). Obesity and being a female were risk factors for long-term arthralgia [200]. Persistent joint pain, including arthritis, tenosynovitis and fibromyalgia were also noted [201]. The joint pain can be so severe it impacts quality of life and the patient′s economic security [202].

#### 2.4.2. Ecology and Transmission Cycles of CHIKV

CHIKV has a worldwide geographic distribution. Phylogenetic analyses reveal the presence of four lineages: West African (WAf), East/Central/South African (ECSA), Indian Ocean (IOL) and Asian. While all lineages are capable of causing human infection, viruses that comprise the IOL (subset of ECSA) and Asian lineages are more associated with large epidemics. Regardless, there is serologic recognition between lineages such that infection (or vaccination) with one lineage can protect against another [203].

CHIKV originated in sub-Saharan Africa where it is maintained in two distinct enzootic transmission cycles between nonhuman primates and forest-dwelling *Aedes* mosquitoes. Here, the WAf and ESCA lineages of CHIKV are vectored by *Ae. furcifer*, *Ae. taylori*, *Ae. luteocephalus*, *Ae. dalzeili*, *Ae. africanus*, Ae. *neoafricanus* and *Ae. cordellieri* in Senegal, Ivory Coast, Uganda, Central African Republic and South Africa [204,205,206,207,208]. Nonhuman primates have been implicated as the principal reservoir for CHIKV based upon both serology and viral isolations from several species. In Senegal, *Chlorocebus sabaeus* (African green monkeys), *Galago senegalensis* (bushbabys), *Cercopithecus aethiops* (guenons), *Papio papio* (Guinea baboons) and *Erythrocebus patas* (patas monkeys) were CHIKV positive confirmed by viral isolation [204]. The transmission cycle appears to peak every 3–4 years, likely coinciding with the birth of new naïve primates [206]. Humans become infected from spillover events from this sylvatic cycle. Urban transmission has not been a characteristic of CHIKV infections in Africa.

Outside of Africa, however, anthropophilic *Ae. aegypti* and *Ae. albopictus* are the main vectors for urban transmission (reviewed in [191]). These mosquito species are well-adapted to live alongside humans and are capable of transmitting many pathogens of human importance, including dengue and Zika viruses. Moreover, the widespread geographic distribution of these mosquito vectors has made them ideal vectors for explosive outbreaks of CHIKV. This is particularly when modern human travel methodologies are taken into consideration, as an individual infected during an outbreak originating in one part of the world can easily transport the virus to any other locale with competent *Ae. aegypti* and/or *Ae. albopictus* where an outbreak can be initiated. Asian and IOL lineage viruses in Asia, India and the Americas are primarily associated with this urban spread and epidemics.

#### 2.4.3. History of Outbreaks

The first cases of CHIKV infection were identified in Africa and spread to Asia shortly thereafter. Between 1952–1953, a febrile illness with sudden onset and sharp crippling pain was described in present day Tanzania. During this time, an isolate was made from both patients and mosquito species thereby confirming CHIKV infection [209]. Throughout the 1960s and 1970s, infections were observed in South Africa, the Democratic Republic of Congo, Senegal, Uganda, Nigeria, Angola, Central African Republic, Ivory Coast and Cameroon [209,210]. The first large urban outbreak of CHIKV was observed in Thailand starting in 1960; this outbreak also incriminated the *Ae. aegypti* mosquito as the predominant urban vector [210,211,212]. Phylogenetic analyses demonstrated that viruses isolated during this epidemic clustered within the Asian lineage, marking the spread of a new virulent epidemic strain of CHIKV [210]. Infections continued into the 1980s and 1990s across multiple African and Asian countries from WAf, ECSA and Asian lineages.

In 2004, CHIKV spread from Kenya to Comoros and ultimately to the island of La Réunion off the coast of Madagascar in the Indian Ocean in 2015. Here, a unique mutation within the E1 protein would significantly alter urban transmission cycles. This E1 A226V mutation allows for mosquito midgut epithelial cells to be more efficiently infected, thereby increasing *Ae. albopictus′* ability to transmit CHIKV significantly [213,214]. The result was increased vectorial capacity leading to enhanced CHIKV spread. Between 2005 and 2006, more than 244,000 cases and 203 deaths would be counted [198]. The La Réunion (LR) isolate, a commonly used strain for experimental infections and vaccine efficacy testing, was isolated during this epidemic. Phylogenetic analyses would place this LR strain in the IOL lineage, a subgroup of the ECSA lineage. This strain would subsequently spread to other islands in the Indian Ocean including Madagascar, India, Sri Lanka, Cameroon, Gabon and Congo. Travel-associated infections were identified in multiple European countries [210] and local transmission was observed in Italy in 2007 [215]. By 2008, CHIKV outbreaks were observed in Malaysia [216], Singapore [217], Thailand [218] and China [219].

Considering the outbreaks of Africa and Asia, experts were surprised that CHIKV had not initiated autochthonous transmission in the Americas, given that the *Ae. aegypti* and *Ae. albopictus* in the Americas were suitable vectors for urban transmission. In 2013, however, a CHIKV isolate belonging to the Asian lineage was identified in the Caribbean. The virus quickly spread to neighboring islands and into South America. Brazil noted its first case in 2014. More than one million cases have been reported to PAHO since [192]. Around 2015, another introduction of an ECSA lineage virus was noted in Brazil with sustained autochthonous transmission, proving that ECSA stains were capable of urban transmission outside of Africa and introduction of new strains continues to be a threat [220,221]. The states in the northeast have been the hardest hit [222,223]. In 2016 and 2017, the number of confirmed CHIKF cases totaled 261,000 and 171,000, respectively [223]. After this initial introduction, CHIKV spread across the South and Central America spreading as far north as Mexico. The majority of cases in the United States were travel related though some might have been locally acquired [224,225]. Interestingly, CHIKV has not initiated an enzootic cycle in the Americas and is an active area of investigation since the mosquito and nonhuman primate species capable of harboring infection are present in the Amazon [226,227].

#### 2.4.4. Potential for Future Outbreaks

The chance of CHIKV continuing to cause outbreaks is high. Since humans are amplifying hosts, a few infected individuals in the context of competent mosquito vectors can potentially initiate an epidemic. Despite millions of infections to date, it is highly unlikely seroconversion in the population has reached levels to sustain herd immunity. No vaccine is currently available to halt transmission, although many candidates are in clinical trials, none are commercially available. Moreover, IOL strains in Asia have already been introduced into other areas of the world multiple times with little reason to suggest it will not happen again in the future. Without a worldwide and sustained vector eradication program, new epidemics are all but certain. Such eradication efforts have had success in the past with *Ae. aegypti* in the Americas, but failure to maintain them and prevent re-introductions leads to re-infestation [228]. Widespread vector control, therefore, cannot be the solution to prevent CHIKV spread. Furthermore, CHIKV has already demonstrated the ability to mutate to take advantage of new vectors. Currently, Asian lineage viruses do not harbor the A226V mutation, thereby limiting their spread by *Ae. albopictus*. However, other point and/or epistatic mutations may occur which could allow for efficient spread by *Ae albopictus*, thereby resulting in enhanced spread. The only methods that will work to prevent future outbreaks will be the widespread deployment of a vaccine that can block CHIKV viremia in vaccinated individuals, thereby halting the spread of virus to feeding mosquitoes.

### 2.5. Mayaro Virus (MAYV)

#### 2.5.1. Disease

Mayaro virus is the etiologic agent Mayaro fever (MAYF). After infection, MAYV causes a transient viremia which lasts 3–4 days and has an incubation period between 7 and 12 days until signs and symptoms become apparent [229]. MAYF is a disease characterized by nonspecific and self-limited sign and symptoms including fever, myalgia, arthralgia and skin rash [230] Arthralgia, headache and fever are the most common clinical manifestations and are present in the majority of symptomatic patients [231]. Rash is observed in about two thirds of the symptomatic patients and only lasts for a few days. Other signs and symptoms include chills, headache, myalgia, eye pain, diarrhea, vomiting and lymphadenopathy. Leukopenia is also commonly observed in patients. Similar to other arthralgic alphaviral diseases, MAYF is currently believed to be a self-limiting disease lasting approximately a week. However, arthralgia can persist for several weeks to months after primary infection [232]. MAYF can also produce more severe complications, including neurological complications, myocarditis and death [229]. The case-fatality in humans is not well-known as the small outbreaks do not allow for an accurate estimate [230]. There are no approved vaccines to prevent MAYF, although several experimental vaccines have been tested in animal models [233,234,235]. There are also no treatments for this disease. Furthermore, MAYF occurs in areas hyperendemic for other arboviral diseases with similar clinical presentations and a confirmation for MAYV infection is not always achieved, leading to possible underreporting of the disease [232].

#### 2.5.2. Ecology and Transmission Cycles of MAYV

The MAYV enzootic cycle consists of mosquitoes of the genus *Haemagogus*, which are considered the primary vector, and several mammalian species, primarily nonhuman primates [236]. More research is needed to determine whether other vectors, host and reservoirs could play a role. *Haemagogus* spp. mosquitoes found to transmit MAYV during its largest outbreak in Belterra, Brazil [237]. Although mosquitoes of the genus *Aedes* can also be productively infected and shed virus in their saliva in laboratory settings [238,239], MAYV was only isolated once from these mosquitoes [240], and human transmission was not observed. Long and collaborators found that at least 5.34 log_10_ PFU/ ml can be found in the blood of infected humans after testing the blood of 25 symptomatic patients [239]. These titers are borderline sufficient for infection of *Aedes* mosquitoes in a laboratory setting [239], and more research needs to be done to accurately determine peak viremia. The fact that no urban outbreaks of MAYV have been reported suggests that these urban and peri-urban *Aedes* mosquitoes likely do not play a major role as vectors.

#### 2.5.3. History of Outbreaks

There are many documented human cases of MAYV infection, however, few large scale outbreaks in the past 66 years [241]. MAYV circulation has been reported only in the Americas, mainly in Brazil, Peru, and Bolivia [241]. Additionally, reports of infections are based on serological tests, which may display a high level of cross-reactivity with antibodies against other endemic alphaviruses [229]. Although the first human case of MAYV was first isolated in the Mayaro county, southwest of Trinidad and Tobago, in 1954 [242], the first registered outbreak occurred in Pará state, Brazil, in 1955. Six strains of MAYV were isolated from blood samples collected from febrile patients [243]. In the same year, an outbreak of “jungle fever” occurred in Bolivia, and MAYV was identified as probable causative agent of 10–15% of 192 cases [244]. Later, nearly 20% of the population (more than 4000 inhabitants) of a rural village in Pará state, Brazil, were infected with MAYV during an outbreak between December 1977 and June 1978 [245]. In another rural community in Pará state, an outbreak of a dengue-like illness was reported in early 2008. One hundred and five patients were examined and 36 (34%) of them had a positive anti-MAYV IgM titer and three MAYV isolations were made [246]. A MAYV outbreak was also registered in the Portuguesa state, west of Venezuela in 2010. Nineteen of 77 cases reported were confirmed as seropositive and six MAYV strains were isolated [247]. Other outbreaks occurred in Brazil in 1981, 1991 and 2015 with no detailed information [241,248]. Finally, there are individual case reports of human infections, virus isolation from mosquitoes, and seroprevalence studies conducted in countries of the Americas that indicate MAYV is probably circulating in several regions of the continent and, therefore, cases of infection may be long underreported [229,241,248].

#### 2.5.4. Potential Future Outbreaks

MAYV has the potential to become a public health issue in the future, although this potential hinges on the virus′ ability to efficiently infect and be transmitted by urban mosquitoes such as *Ae. aegypti*, which has to date not been demonstrated in nature [138,229]. Indeed, many have predicted MAYV may be the next arbovirus to cause a large epidemic [231,248,249]. However, there are several gaps in knowledge that need to be addressed in order to improve the understanding of this virus and the capacity to predict and prevent MAYV outbreaks [241]. It is known the virus is transmitted by *Haemagogus spp*. in the Americas [241], but further vector ecology and surveillance studies are essential to analyze the presence of these mosquitoes and other potential vectors and their capacity to introduce MAYV into non-endemic areas. Imported cases in urban centers with the circulation of vector mosquitoes may play a role in potentially spreading the virus into these areas [250]. It is also possible that MAYV could adapt to replicate in *Aedes* mosquitoes causing large urban outbreaks. MAYV is present in South America [231,247,251,252] and it may also be present to a lesser extent in Central [130,253] and North America [130,254], and it is known to cause small outbreaks in areas close to forests. Adaptation to an anthropophilic mosquito vector could more readily permit sustained urban transmission. Additionally, there is a high antigenic cross-reactivity between MAYV and other viruses of the Semliki forest virus serocomplex [241]. This may result in poor accuracy for serological assays and potential enhancement or protection of infection in regions displaying co-circulation of these alphaviruses. The former issue needs to be overcome to improve our capacity to evaluate the circulation of MAYV by serological studies and also accurately diagnose human cases, as these are essential measures to develop a better response to potential MAYV outbreaks. The latter issue has implications regarding disease severity in most of the affected regions and, consequentially, to vaccine development, which is the best prevention method that is still currently unavailable [229,255]. No vaccine currently exists against MAYF, but experimental work in mice suggests that vaccination with attenuated CHIKV might confer some protection against MAYV infection and disease [256].

### 2.6. Sindbis Virus (SINV)

#### 2.6.1. Disease

The Sindbis virus is the causative agent of Sindbis disease and belongs to the arthritogenic group of alphaviruses [190,257]. The disease caused by the SINV infection is named differently according to the region affected: Pogosta disease (Finland), Karelian fever (Russia), and Ockelbo disease (Sweden) [257]. There are five different genotypes (SINV-I to SINV-V) comprising the SINV complex and they display significant genetic variability among them, reaching 28.2% on the nucleotide level and 22.2% on the amino acid level [258]. These strains can be found in specific regions of Africa, Europe, Asia, and Oceania and their location is probably related to the dissemination by migratory birds [258,259]. Additionally, human outbreaks are primarily associated with SINV-I [260]. Despite this wide distribution of the SINV, the most affected regions are South Africa and countries in Northern Europe [257,260].

The SINV infection is usually self-limited or subclinical and, therefore, largely undiagnosed [261,262,263,264]. Serological surveys in Finland [264] and Sweden [261] indicate that as much as 95% of all SINV infections may not be diagnosed. After an incubation period of four days (2–10 days range) approximately [265], patients develop some signs and symptoms that are similar to other arthritogenic alphaviruses. The main symptoms experienced by patients with the disease are rash, arthralgia, and fever [266]. However, reports show that a wider range of symptoms is observed. Frequent symptoms and signs are papular rash, arthritis or arthralgia, fatigue, muscle pain, headache, fever, itching, and upper respiratory symptoms. Nausea, dizziness, enlarged lymph nodes, diarrhea, and photophobia have also been shown, though less frequently [265,267]. Most of the patients recover completely from muscle and debilitating joint pain over time [257], with a median duration of nine days, though it can last for up to 80 days [265]. A small percentage of patients require hospitalization, but severe complications are rare and fatalities have not been reported [257,265]. The most striking impact of the SINV infection is that several patients can experience joint symptoms for months or even years [265,267]. Kurkela et al. [268] investigated a cohort of patients in Finland and found that 24.5% of the patients experienced joint manifestations three years after SINV infection. A similar observation was made by Niklasson and Espmark in the 1980s, when 27 out of 86 patients (31.4%) complained about arthralgia 3–4 years after SINV infection [269]. Arthralgia lasting for 5–6 years has also been described [270]. Since these symptoms can heavily impact the life of the affected patients, the economic and public health burden can be considerable in endemic areas [257]. Additionally, patients who have joint tissue damage as an underlying condition may be at increased risk of developing SINV infection-associated severe symptoms [265].

#### 2.6.2. Ecology and Transmission Cycles of SINV

The SINV is a mosquito-borne avian virus, similar to some members of the alphaviruses [259]. Hence, the SINV transmission involves an enzootic cycle between primary hosts (birds) by mosquitoes and an eventual spillover to humans, also via mosquitoes, may result in disease development [257]. Indeed, SINV was first isolated from mosquitoes and a juvenile hooded crow (*Corvus corone sardonius*) during an epidemic in Cairo, Egypt [271]. SINV replicates within mosquitoes, birds, and humans, however, the latter does not produce the required viremia for mosquitoes to become infected during a blood meal. Hence, humans are considered dead-end hosts of SINV transmission, which precludes a human–mosquito–human urban cycle [259,272,273].

Mosquitoes from the widely distributed Culex genus have been implicated as main vectors in different affected regions [258,274]. Overall, most studies identify *Cx. torrentium* and *Cx. unvittatus* as main enzootic vectors in Northern Europe [257] and South Africa/Australia [274], respectively. Further, the virus has also been isolated from *Cs. morsitans* and *Aedes* (*Ae*.) *cinereus* in Sweden [275,276], *Ochlerotatus* spp. in Finland [277], and *Aedes* spp. in Russia [278]. Evidence of transmission from birds to humans implicates *Ae. cinereus* as bridge vector of SINV [260,279].

Birds serve as both reservoirs and amplifying hosts of the virus of SINV. Studies highlight migratory birds in the redistribution of SINV from enzootic regions into non-endemic areas, as the differences between genotypes of SINV are associated with the geographic patterns of these birds [258,280]. Additionally, birds might have been spreading SINV mainly through north–south axis migratory routes, rather than east–west axis flyways [257]. Although studies of experimental infection of different birds and their potential role as SINV amplifying hosts are currently scarce [281], studies of anti-SINV antibodies detection and viral isolation from birds collected in the wild suggest a relatively wide spectrum of potential amplifying hosts of SINV. Lundström et al. [33] detected neutralizing antibodies against SINV in birds from three orders, Anseriformes (waterfowl), Galliformes, and Passeriformes in Sweden. Fourteen species of these orders can also be experimentally infected with SINV, producing sufficient viremia titers to infect enzootic cycle mosquitoes [282]. Furthermore, increased seropositivity in fieldfares (*Turdus pilaris*), redwings (*Turdus iliacus*), and of song thrushes (*Turdus philomelos*) suggests that these birds, which are common in Northern and Central Europe, might be the main amplification hosts in Sweden [283,284]. A study in Finland detected antibodies anti-SINV mainly in two *Tetrao* spp., black grouse (*Tetrao tetrix*) and capercaillies (*Tetrao urogallus*) [264]. The SINV has also been isolated from hooded crow (*Corvus corone cornix*) [281] and from a wild, common wood pigeon (*Columba palumbus*) in Germany, which is also common in Central Europe [285] and others [258]. Therefore, monitoring studies of birds, mosquitoes, and other potential hosts are warranted in order to continue evaluating the risk of SINV outbreaks.

#### 2.6.3. History of Outbreaks

The SINV was first isolated from mosquitoes during an epidemic in the village of Sindbis, close to Cairo, Egypt in 1952 [271]. Later, the virus was also isolated from skin lesion samples from a woman infected in South Africa [286]. Noticeably, SINV outbreaks in the African continent have been reported by South Africa only [257]. Later in 1963, an outbreak of similar cases as the women whom the virus was isolated occurred in South Africa and 14 out of 21 (66.6%) patients were serologically diagnosed with SINV infection [287].

In 1965, neutralizing antibodies against SINV were detected in children from a WNV-endemic area in Israel. The analyzed population showed a 6.51% (23/353) seropositive rate [288]. SINV neutralizing antibodies were also found in Egypt in 1967 [289]. Serological testing following the febrile illness of a child in Australia in 1969 also showed evidence of SINV infection [290]. Serological studies have indicated the presence of SINV in European regions as well [257,291,292]. Furthermore, Northern Europe emerged as a focus of SINV infection in Sweden in 1967 [293] and Finland in 1974 [264]. SINV infections have been recorded yearly since in this region [257].

Since 1974, outbreaks in Finland occur in approximate seven-year cycles without a directly implicated cause [264]. In the outbreak reported in 1981, several patients presented rash (88%) and joint symptoms (99%) [294]. In 1989, a serological survey of the affected regions revealed a prevalence of 2.7−5.7% of anti-SINV antibodies, depending on the analyzed location [295]. A more recent and larger study revealed a seroprevalence of 9−17% in regions of Finland in the early 2000s [296]. Additionally, Kurkela and collaborators [297] were able to isolate the causative agent of the SINV outbreak in 2002 for the first time in Finland. In addition to the major epidemics in 1995 and 2002, when 1310 and 597 laboratory-confirmed SINV cases were identified, respectively, the predicted outbreak of 2009 only manifested in 105 cases that were diagnosed that year [298]. However, there was a peak in diagnosed cases in 2012, when almost twice the number of cases were reported than occurred in 2009 [257,260].

In Sweden, after the first registered outbreak in 1967, many cases of SINV infection were reported in 1981 [293], and preceded another outbreak in 1982 [299], when 36 and 65 cases were serologically confirmed, respectively [270]. From 1983 to 1988, 141 cases were identified with the highest number (40%) seen in 1988 [261]. The 1995 outbreak in Finland was also identified in Sweden but with fewer cases [260]. The last reported outbreak in Sweden occurred in 2013 in a previously non-endemic area in the northern region of the country [275].

South Africa is another country significantly affected by SINV; however, epidemiological data and public health burden in South Africa are still quite vague [262]. The simultaneous outbreak of WNV and SINV in 1974 resulted in thousands of cases of infection by these arboviruses, which was the largest outbreak ever registered at that time [264,300]. Later, an outbreak between late 1983 and early 1984 resulted in hundreds of cases clinically diagnosed as SINV infection. Among these, 28 were serologically confirmed infections by SINV [301]. A retrospective study of suspected arbovirus infections has indicated that an outbreak of SINV and Rift Valley virus occurred in 2010, which was preceded by the yearly detection of a small number of SINV infection cases between 2006–2009. While a total of 21 samples were seropositive for SINV from 2006 to 2009, 208 cases were anti-SINV IgM positive in 2010 [262].

#### 2.6.4. Potential for Future Outbreaks

Finland has reported more SINV cases than other areas but this may be due more to reporting than actual prevalence as required reporting was instituted in 1995 [298]. Since this year, the number of cases in Finland increased dramatically in comparison to Sweden, for example, which did not adopt this measure. Notably, the number of cases in Finland was 33 times higher than Sweden′s from 1995 to 2012 [260]. Since 1974, SINV outbreaks have occurred every seven years in Finland without a firm underlying reason [264]. One hypothesis is the susceptibility of immunologically naïve amplifying hosts; the population cycles of grouse overlap with SINV outbreaks years [264]. Therefore, these observations suggest that forecasting future outbreaks based on this seven-year cycle may not be accurate and objective markers of SINV activity. Interestingly, Lundström et al. [260] showed increased detection of SINV-I in *Ae. cinereus* and *Cx. torrentium/pipiens* mosquitoes collected during the late mosquito season of an outbreak year in comparison to pre- and post-outbreak years. These findings suggest, at least for Northern European countries, that the surveillance of SINV prevalence in bridge and enzootic vectors during late mosquito season may constitute a good marker to predict outbreaks caused by this virus [260].

Virus circulation outside of Europe has been detected primarily based upon virus isolation and serology in native populations rather than outbreaks of Sindbis disease. SINV has been isolated from mosquitoes in Kenya [302]. South Africa has reported infections but underreporting is highly likely as Sindbis disease may be attributed to other illnesses with similar symptoms [259]. SINV has been circulating within Australia for at least 33 years in different locations of the country based upon phylogenetic analysis [280]. However, there are only two reports of human cases in Australia, a child in 1969 [290] and a man in 1980 [303]. In Israel, the virus seems to be constantly circulating in the country recently, as SINV-I was detected in mosquito pools collected during the period of 2004–2006 and 2013–2015 [304]. Also, a serological survey conducted from 2011 to 2014 demonstrated a prevalence of IgG anti-SINV of 1.9−3.6% [305]. In Germany, Jöst et al. showed [306] the presence of SINV in mosquitoes collected in the southwest region of the country in 2009. The virus was isolated from *Anopheles maculipennis* and the enzootic vectors *Cx. torrentium* and *Cx. pipiens*. Phylogenetic analysis revealed that the strains isolated were linked to Swedish strains and related to human cases in Sweden [306]. In addition, IgG anti-SINV was detected in four healthy blood donors (from 3389 samples), though no specific IgG, IgM or SINV RNA was detected in patients with suspected infection from an area with high SINV infection in mosquitoes [307]. It seems improbable that SINV outbreaks may occur in the analyzed area in Germany when these findings are considered along with the lack of evidence of the presence of the SINV bridge vectors. Still, future studies are necessary to provide information about the risk of human SINV infection, not only in Germany but also in other regions where mosquitoes infected with this virus can be found [306].

Similar to other arthropod-transmitted virus, SINV outbreaks could occur when enzootic vectors and reservoir birds are infected with SINV-I and the virus is transmitted from birds to humans by bridge-vector mosquitoes [260]. Since countries in which these hosts can be found are at risk of human infections, mosquito and viral surveillance in such regions are recommended as public health measures to detect potential outbreaks of SINV infection and reduce their impact on the population. Nonetheless, as potential SINV vector and host populations may reflect on the geographic limitation of SINV outbreaks, this needs to be further investigated [259].

### 2.7. Ross River Virus (RRV)

#### 2.7.1. Disease

Ross River virus causes Ross River virus disease (RRVD) in Papua New Guinea and is the most common arboviral disease in Australia [308,309,310,311]. Patients experience a variety of symptoms ranging from subclinical to serious [311]. Approximately 55–75% of human RRV infections are asymptomatic [309,310]. Disease usually appears for 7–9 days, with the period of incubation varying from 3 to 21 days [309,312]. The signs and symptoms of RRVD are similar to other related alphaviruses [313], including maculopapular rash, rheumatic manifestations, fatigue, myalgia, fever, and lymphadenopathy [308,309]. Less frequently, patients can also present splenomegaly, hematuria, glomerulonephritis, paresthesia, headache, neck stiffness, and photophobia. Meningitis or encephalitis has also been reported, although this may be incidental to RRV infection [309]. While the common symptoms may last for a few days or weeks [311,314], rheumatic symptoms may last for up to six months, according to currently available case reports [309,315,316]. No fatality has been directly linked to RRV infection [309,317].

#### 2.7.2. Ecology and Transmission Cycles of RRV

RRV is maintained in nature within an enzootic cycle between vertebrate hosts, mainly macropod marsupials, and mosquito vectors [309,311]. Despite the key role of marsupials, the ecology of RRV is actually quite complex, since there is a large number of other potential vertebrate hosts [309,314], such as non-macropod marsupials, bats, and horses [318]. Additionally, evidence suggests that human–mosquito–human transmission may occur as well, as outbreaks have occurred in regions where suitable nonhuman vertebrate hosts are absent [309,319,320]. The RRV was first isolated in 1959, Australia, from a pool of female *Ae. vigilax* mosquitoes [321]. Notably, *Ae. vigilax* and *Ae. camptorhynchus* are the most important vectors in coastal regions, while the principal vectors in the inland are *Cx. annulirostris* and *Coquillettidia linealis* [318,322]. The virus has also been isolated from several other *Aedes* species and other genera as well, with some species proven to be able to transmit RRV by vector competence studies [317,318]. Furthermore, it seems that other species may play a role of transmission in specific regions, given the diverse range of vector habitats in these locations [318,323]. Therefore, there is a singular complexity in the ecological transmission of RRV.

#### 2.7.3. History of Outbreaks

RRV was isolated from mosquitoes in 1959 [321], but reports of RRV outbreaks have occurred in Australia since 1928. Several outbreaks were also registered in Australia and Papua New Guinea during the Second World War and in 1956 [309]. The last outbreak was particularly important, as it was suggested that the causative agent of this outbreak was probably a new member of the group A of arboviruses [324]. This new member was found to be the “T48” virus [321], named RRV a few years later. The first isolation of RRV from human samples was done in 1971, from an aboriginal child in Australia [325].

RRV spread to the South Pacific in 1979, subsequently causing important outbreaks in Fiji [326], Cook Islands [327], and American Samoa [319]. There have been no registered outbreaks in the Pacific Island Countries and Territories since these events, however serological studies indicated that the virus is still circulating in some of these countries recently [310]. In Australia, outbreaks have been registered almost on a yearly basis, increasing in frequency and individuals affected in recent decades [328]. However, the diagnosis and reporting systems have improved from 1990 to recent days, hence case numbers recorded before previously may not be adequately comparable [318]. Recently, around 5000 cases of RRV infection were reported yearly in the country, from 1991 to 2001 [329]. From 2000 to 2016, the mean of reported cases was 4541 per year [330], with emphasis on the 2014–2015 season, when 10,074 RRV cases were reported and constituted the highest number of RRV notifications ever recorded [330,331].

#### 2.7.4. Potential for Future Outbreaks

There are no antiviral drugs or specific therapeutics against RRV infection [311]. Vaccination studies are being conducted and one vaccine candidate has even completed phase III clinical trials, but it is still unlicensed, hence, currently unavailable to the general population [314]. Therefore, new outbreaks of RRVD are a continuous threat in Australia [318], and the virus may also pose a threat to other regions where conditions to enable its propagation are present [309]. According to Harley and collaborators [309], the conditions that determine the human risk for RRVD are adequate populations of reservoir hosts, vector mosquitoes, and climate conditions for transmission. Since evidence suggests that RRV may be a multi-host pathogen, this must be investigated in-depth across different ecosystems [332]. Addressing this issue is definitely challenging, though of utter importance to improve our knowledge about the RRV dynamics and its epidemic potential. Additionally, among climate variables, local rainfall is probably the most important factor that supports RRV spreading in endemic locations, as increased rainfall may increase the abundance and survival of adult vector mosquitoes [328,330]. However, given the high complexity of RRV ecology, it is imperative to analyze these factors at the local level in order to better predict and control RRVD epidemics [328]. Finally, long term studies of RRVD on affected patients, as well as potential effects of previous conditions to induce severe forms of disease by RRV infection are warranted to further explore the impact of this infection.

## 3. Future Threats and Considerations

**Figure 1:**
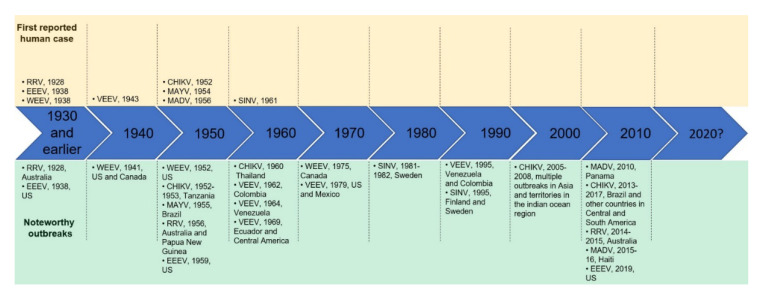
*Alphavirus* outbreak timeline. History of human alphavirus infections based on first reported human infection (Above) and important human outbreaks (Below) for each virus.

In the past century, several arthropod-borne viruses have emerged and/or re-emerged and have been associated with significant morbidity and mortality [34,138,333] (Summarized in Figure 1). Among these agents, viruses within the genus *Alphavirus* represent a significant risk to both human and animal health. In particular, CHIKV has demonstrated the substantial risk posed by Alphaviruses in the contemporary age. The single mutation, E1-A226V significantly increased the fitness of IOL strains of CHIKV in the peri-urban vector *Ae. albopictus*, a vector known for its ability to survive in colder environments than *Ae. aegypti*, allowing for the geographic expansion of CHIKV, and displacement of Asian lineage strains in some areas [214,334,335,336]. The Asian lineage itself expanded the geographic range of CHIKV via a series of outbreaks in the Americas beginning in 2013, which are estimated to have infected over 2.9 million individuals [337,338]. Given the backdrop of climate change, and human activities including global travel and commerce, destruction and encroachment of local ecologies, the emergence/reemergence of Alphaviruses and other arboviruses is not only possible but likely in coming years [136,138,339]. With the exception of WEEV [188], the alphaviruses highlighted here continue to have significant potential to emerge/reemerge and cause significant human and veterinary disease. Moreover, the lack of critical health care infrastructure and antiviral measures such as vaccines and antivirals remains a significant hindrance for treating future outbreaks. Therefore, enhanced virus surveillance and mosquito abatement will be critical tools to stemming potential epizootics.
